# The Pain Management of Trauma Patients in the Emergency Department

**DOI:** 10.3390/jcm12093289

**Published:** 2023-05-05

**Authors:** Andrea Fabbri, Antonio Voza, Alessandro Riccardi, Sossio Serra, Fabio De Iaco

**Affiliations:** 1 Emergency Department, AUSL Romagna, Presidio Ospedaliero Morgagni-Pierantoni, 47121 Forlì, Italy; 2 Department of Biomedical Sciences, IRCCS Humanitas Research Hospital, 20089 Milano, Italy; 3 Unit of Emergency Medicine, Ospedale San Paolo, 17121 Savona, Italy; 4 Emergency Department, AUSL Romagna, Ospedale M. Bufalini, 47521 Cesena, Italy; 5 Struttura Complessa di Medicina di Emergenza Urgenza, Ospedale Maria Vittoria, ASL Città di Torino, 10144 Torino, Italy

**Keywords:** pain relief, multimodal approach, analgesic dose, emergency department, main drugs

## Abstract

The vast majority of injured patients suffer from pain. Systematic assessment of pain on admission to the emergency department (ED) is a cornerstone of translating the best treatment strategies for patient care into practice. Pain must be measured with severity scales that are validated in clinical practice, including for specific populations (such as children and older adults). Although primary care ED of trauma patients focuses on resuscitation, diagnosis and treatment, pain assessment and management remains a critical element as professionals are not prepared to provide effective and early therapy. To date, most EDs have pain assessment and management protocols that take into account the patient’s hemodynamic status and clinical condition and give preference to non-pharmacological approaches where possible. When selecting medications, the focus is on those that are least disruptive to hemodynamic status. Pain relief may still be necessary in hemodynamically unstable patients, but caution should be exercised, especially when using opioids, as absorption may be impaired or shock may be exacerbated. The analgesic dose of ketamine is certainly an attractive option. Fentanyl is clearly superior to other opioids in initial resuscitation and treatment as it has minimal effects on hemodynamic status and does not cause central nervous system depression. Inhaled analgesia techniques and ultrasound-guided nerve blocks are also increasingly effective solutions. A multimodal pain approach, which involves the use of two or more drugs with different mechanisms of action, plays an important role in the relief of trauma pain. All EDs must have policies and promote the adoption of procedures that use multimodal strategies for effective pain management in all injured patients.

## 1. Introduction

Pain is known to impair respiratory function, immune response and wound healing and worsen patient outcome by increasing metabolic demand in patients with severe trauma. Inadequate treatment of acute pain after trauma delays return to work, impairs quality of life and increases the risk of complications such as post-traumatic stress disorder [1].

There is still an unmet need in the treatment of trauma pain that spans the patient’s life course. Poor pain control is the primary risk factor for developing chronic pain syndrome, a very disabling condition. It is estimated that almost two-thirds of patients report at least moderate pain 12 months after injury, and three out of four patients report that pain interferes with activities of daily living, work, and cognitive, psychological and emotional disturbances, particularly with a decrease in self-esteem and the development of depression [2]. These consequences increase the risk of a feeling of distress that in turn feeds the pain itself and triggers a vicious cycle of trauma-pain-stress-feedback.

In pain management, the main goal is a tolerable level of pain, i.e., a level of pain that is acceptable to the patient and allows them to function at a minimum.

An estimated 38 million people in Europe visit EDs each year because of a traumatic event, of whom more than 5 million are hospitalized [3]. Pain is one of the main symptoms in at least 90% of cases [4], but inadequate relief from trauma pain is also reported by patients [5,6]. In a multicenter study [7] conducted in the United States and Canada, 74% of patients were discharged from EDs with moderate to severe pain; this is similar to rates reported in European studies [6].

Studies have shown that up to two-thirds of trauma patients can wait up to an hour for pain relief at EDs, and even if they do receive pain-relieving medication, it is only effective in proportion to the degree of pain [8]. 

The lasting consequences of inadequately treated acute pain are thought to be multiple and severe, both in the short and long term. These consequences include increased risk of infection, decreased comfort and progression to chronic pain syndrome, a particularly disabling condition that has significant economic and social consequences [9].

In Europe, the treatment of trauma pain in the prehospital setting and in the ED is largely similar and consists mainly of paracetamol, non-steroidal anti-inflammatory drugs (NSAIDs), nitrous oxide (N_2_O) and opioids [4,10,11]. The current use of these analgesics can be considered inadequate. For example, prospective data from Norwegian and Italian EDs showed that only 14% and 32% of patients with moderate to severe pain received analgesia, respectively [11,12].

The lack of effective pain management not only affects the patient, but also the entire ED environment, as healthcare providers are expected to manage increasingly severe pain, which in turn has resource implications. There appears to be an unmet need for safe, timely and effective management of trauma pain in the emergency setting. However, there are barriers to effective management of pain in the ED, largely due to the lack of effective national guidelines for pain management, delayed or absent pain assessment, reluctance to use opioid analgesia and delay in the administration of analgesia [5,6,13].

All these difficulties lead to inadequate and ineffective results. There is an unmet need for new forms of analgesia, for wider use of available analgesics that overcome some of the limitations associated with the various treatment options and for the development of pain management protocols.

## 2. Pain Severity and Treatment

In the treatment of acute pain in injured patients, drugs should be used that are quick and easy to administer, have a very short half-life, are highly effective and have minimal side effects. The choice of drugs and appropriate methods of administration should consider the response to and the need for continuous analgesia throughout the course of the disease and in the phases of rehabilitation.

Common analgesics used in ED settings in Europe include opioids, N_2_O, paracetamol and NSAIDs [6,13]. The types of analgesic used are tailored in relation to the type of injury, pain severity or triage system in the ED [10,13,14].

Regional blocks, for example local anesthesia and peripheral nerve blocks, may also be administered in the treatment of trauma pain [15,16]. These treatments may reduce the need for rescue/additional analgesic treatment [17]. Although not a common theme in the literature identified in this search, non-pharmacological approaches also play an important role in improving trauma pain; this includes immobilizing limbs and applying dressings or ice packs, and these approaches may be used in conjunction with drug therapy [18]. Some treatment options have limitations that may hinder effective pain relief in emergency settings.

### 2.1. Pain Assessment

Pain assessment is a complicated process, as using an instrument with a one-dimensional measurement scale may not accurately reflect the multidimensional nature of pain. In any case, in order to assess the effectiveness of treatment and the possible need to administer further medication, it is useful to be able to refer back to a measurement system without changing the scale chosen for the initial pain assessment. The scientific community agrees on the use of one-dimensional measurement scales that relate pain intensity to the type of treatment to be applied (Table 1). In the pediatric pain trauma, the recommendations allow for the use of the FLACC [19,20], Wong-Baker [21] and NRS [22] algometric scales, based on the age of the child, as indicated by literature, and the administration of analgesics, based on protocols shared by the team, if the score obtained is >4 [22].

The assessment of a patient with acute trauma pain can be complex due to the patient’s age, emotional state (anxiety, psychomotor discomfort) and/or change in state of consciousness, for example, in relation to the state of consciousness or the patient’s age. For example, in a patient with trauma, pain is classified as mild to moderate if the NRS score is 1 to 3 and the patient responds to paracetamol and/or NSAIDs; moderate to severe if the NSR score is between 4 and 6 and the patient responds to mild opioids and/or NSAIDs and paracetamol; and moderate to severe if the score is between 7 and 10 and the pain responds to treatment with strong opioids and NSAIDs.

### 2.2. Mild to Moderate Trauma Pain 

Paracetamol and/or NSAIDs are often used as first-line therapy for mild to moderate pain; the route of administration is usually oral or intravenous (IV), depending on the patient’s setting and needs. NSAIDs are commonly prescribed in Europe, and include ibuprofen, diclofenac and naproxen [5,6,13]. In a double-blind study, paracetamol was found to be non-inferior to diclofenac as an analgesic for acute, mild musculoskeletal trauma [19]. However, paracetamol does not have the anti-inflammatory properties of NSAIDs.

NSAIDs work by inhibiting cyclooxygenase enzymes (COX-1 and 2), thus inhibiting prostaglandin synthesis; in some studies, they appear to be equally as effective as opioids for acute traumatic pain in adults and children [25,26].

NSAIDs carry substantial risks of gastrointestinal bleeding, acute kidney injury and cardiovascular events, so their use in trauma is limited to cases of mild trauma with a low risk of complications. Most of the risks are associated with long-term use, and risks can vary based on COX selectivity, even among drugs in the same class. Although the primary risk of NSAID therapy is gastrointestinal bleeding, all NSAIDs have antiplatelet activity that contributes to an increased risk of bleeding at any site. The antiplatelet effects occur primarily through inhibition of COX-1; therefore, COX-2 selective NSAIDs (e.g., celecoxib) have a reduced risk of inhibiting platelet aggregation. While there is no high-quality evidence on the use of NSAIDs in the presence of traumatic brain injury, most practitioners avoid the use of both COX-1 and COX-2 inhibitors in this setting [27]. Many practitioners fear that the use of NSAIDs after trauma could impair the healing of wounds and fractures and require caution when used in patients with coagulopathies or risk of stress-related mucosal bleeding [27].

N_2_O is an inhaled, rapid-onset, short-acting analgesic that is often used in emergency situations [28]. N_2_O has been used for many years as an analgesic in prehospital care and EDs, where its short duration of action is well suited for the treatment of acute trauma pain [29].

Metamizole (dipyrone) is a non-opioid analgesic whose use in EDs varies widely across Europe [30]. In some countries (e.g., the UK, Sweden and some non-European countries. including the USA), metamizole is banned due to concerns about myelotoxicity, but its use is widespread in others (e.g., Spain and Germany, based on discussions by the authors) [31]. A systematic review found that further large-scale studies are needed to better understand the risks and benefits of metamizole when compared to other analgesics [32]. 

Weak opioids, such as codeine and tramadol, are also used to treat moderate trauma pain [5,6,33]. Tramadol acts at L-opioid receptors and inhibits the reuptake of serotonin and noradrenaline [34]. Tramadol is not indicated in patients taking serotonergic drugs or in those with underlying seizure disorders [35]. This leads to an atypical analgesic effect when compared to the usual analgesics of this class and to a less severe side effect profile. Typical opioid side effects are rare with the use of tramadol, making this analgesic a useful analgesic option [36].

### 2.3. Severe Trauma Pain 

Opioids provide effective analgesia for severe trauma pain and are available through various routes of administration, including intravenously (IV), intranasally (IN), intra-osseously (IO), subcutaneously (SC) and per os (PO). While morphine is most commonly used in emergency situations for severe pain in Europe, other opioids, such as fentanyl and oxycodone, are also commonly used [5,6]. Due to pharmacokinetic and pharmacodynamic changes with age, opioids should be started at a lower dose, about 25–50% of the dose given to younger patients [37]. 

Opioids modulate pain signaling in the ascending and descending pathways of the brain and spinal cord and at the supraspinal level, similar to endogenous opioid peptide ligands. Administration activates the brain’s reward system within the ventral tegmental area and frontal cortex; therefore, repeated use increases the risk of tolerance and dependence. They very effective drugs for pain relief that function by exploiting their high affinity for mu receptors located in the central nervous system. At the level of the spinal cord, however, they act on specific receptors located in the pre- and postsynaptic synapses in the dorsal horn. At the pre-synaptic level, opiates, coupling with G proteins, decrease the release of specific pain neurotransmitters (i.e., substance P), decreasing their neuronal excitability at the post-synaptic level [38] through the inhibition of cyclic adenosine monophosphate (cAMP) [39].

The physiology of pain may help clarify why opioids are such effective pain killers. The physiology of pain implies that a noxious stimulus originating in the periphery (such as that of a trauma) is transmitted via primary afferents to the dorsal root ganglion and, from there, to the dorsal horn of the spinal cord. From the spinal cord, the pain signal travels along the ascending pain pathways to the spinothalamic tract of the central nervous system. The brain also sends a signal down descending pathways that modulate the incoming signal.

Opioid agents mimic endogenous opioids and work by binding to (have affinity for) opioid receptors G protein 7-transmembrane coupled, thereby activating them (agonist action, intrinsic activity) albeit with individual differences in receptor binding and signal transduction [39]. Opioids therefore inhibit incoming signals along afferent pain pathways or relieve pain by interacting with descending pain pathways. Naturally, responses are individual and vary depending on emotional state, past experiences and genetics [39].

The pharmaco-kinetic and pharmaco-dynamic characteristics of opioids help to explain the differences between available drugs and are used to choose a starting point for management and additional titration in a multimodal approach [40].

In the use of opioids, the relationship between concentration and effect is often variable and unhelpful in predicting both efficacy and adverse effects [40]. The concentration/effect ratio is not useful because the analgesic effect often lags behind the peak concentration. After a bolus dose of morphine, there is no predictable relationship between the plasma concentration of morphine and the analgesic effect over time; however, with fentanyl, the response decreases rapidly with decreasing plasma concentration after bolus doses of fentanyl. These differences can vary greatly due to the lipid solubility of the drug and the percentage of drug ionized at physiological pH.

For example, fentanyl has a higher lipophilicity than alfentanil, and alfentanil is 100 times more soluble than morphine. At a pH of 7.4, fentanyl is less than 10% ionized, alfentanil about 90% and morphine about 20% [41]. Higher lipid solubility and a higher proportion of the drug in the ionized state facilitates passage through the blood-brain barrier and thus the effect on the central nervous system [42]. Various genetic polymorphisms (e.g., poor metabolizers or ultra-rapid metabolizers) may affect opioid metabolism in such a way that they may cause patients to have a lower or higher response than expected. 

Genetic differences in patient response are particularly common with codeine, but can also occur with other opioids [43,44]. For this reason, in the multimodal approach, opioid rotation, i.e., switching from one opioid to another, can be very helpful when a patient does not obtain the desired analgesic benefit from one preparation, as this patient may respond better to another opioid [45].

Concomitant use with other central nervous system depressants (e.g., benzodiazepines, skeletal muscle relaxants, gabapentin, etc.) should be avoided; administering opioids orally is reserved for the phases following the initial phase in order to maintain the continuum of analgesia or in the phases following the initial phase when pain is controlled. Long-acting products (e.g., extended-release preparations and transdermal preparations) are not appropriate for the treatment of acute pain and should only be used in the post-acute phase. Clinicians should initiate opioid tapering, particularly during downward care transitions, with a desired goal of no opioid therapy at hospital discharge in opioid-naïve patients before hospitalization [46].

Clinicians should base their selection of opioid therapies on patient-specific factors such as organ dysfunction (for example, avoidance of morphine in patients with renal impairment) and desired duration of action (for example, fentanyl for premedication in shorter procedures such as chest tubes but morphine or hydromorphone for breakthrough pain).

Specific dosing/tapering regimens for trauma patients vary based on the type of injury, organ dysfunction, operating schedules and many other clinical and demographic factors.

Ketamine, a phencyclidine derivative that acts as a fast-acting N-methyl-d-aspartate (NMDA) antagonist, is particularly effective in the initial stages of treatment of the trauma patient [47]. The usual dose of ketamine in clinical practice for the treatment of acute pain is an intravenous (IV) 0.3- to 0.5-mg/kg bolus with or without infusion (usually at 0.1–0.2 mg/kg per h), depending on the duration of the patient’s required analgesic response [48].

Ketamine is a highly lipophilic substance with rapid distribution and immediate passage through the central nervous system. It has low plasma protein binding (10–50%), an alpha half-life of 2–4 min, a beta half-life of 2–4 h [49] and a large volume of distribution (160–550 L). The liver metabolizes ketamine via cytochromes CYP 2B6 and CYP3A4, producing (R,S)-nor ketamine, which is metabolized to 6-hydroxynorketamine and 5,6-dehydronorketamine [50]. The metabolites have a half-life of up to 3 days and have important analgesic and antidepressant effects. Bioavailability and efficacy vary according to the route of administration: intravenous administration has a bioavailability of 100% and maximum effect is achieved within 1–2 min; intramuscular administration has a bioavailability of 93% with maximum effect within 5 to 10 min [50]; oral administration has a bioavailability of 16–29% and maximum effect is achieved within 20 to 120 min. Oral administration of ketamine is considered less beneficial due to lower bioavailability and a significant first-pass effect in the liver [50].

Ketamine has a complex relationship with opioid receptors. By interacting with central and spinal opioid receptors and N-methyl-D-aspartate (NMDA) receptors, it reduces opioid tolerance, opioid-induced hyperalgesia and central sensitization [51]. Ketamine also activates NMDA receptors, leading to postsynaptic hyperexcitability, central tolerance and sensitization. Ketamine modulates and reduces these effects, as shown with NMDA antagonists such as MK-801 [52]. Ketamine has a downstream effect; it enhances opioid-induced phosphorylation of extracellular signal-regulated kinase 1/2 (ERK 1-2), resulting in a reduction in the amount of opioid required to achieve the desired therapeutic effect (sparing effect). This also contributes to the reduction of adverse events, including respiratory depression and vomiting [53].

The broad therapeutic index, cardiovascular stability and lack of respiratory depression make ketamine attractive for use in the prehospital setting [54,55]. The dissociative effect associated with ketamine also makes it an effective treatment for trauma pain, although safety concerns have been raised regarding psychological manifestations and long-term psychotomimetic effects [56].

Low-dose methoxyflurane, a non-opioid, volatile fluorinated hydrocarbon, is administered via a hand-held inhaler. While the use of methoxyflurane for general anesthesia has been discontinued due to renal safety concerns, administration of sub-anesthetic concentrations over short periods of time is not associated with nephrotoxicity [57]. 

Methoxyflurane has been used in emergency situations in Australia and New Zealand for over 30 years and has recently been approved in some European countries (including Belgium, France, Ireland and the United Kingdom) for the emergency treatment of moderate to severe pain in conscious adults with trauma and associated pain [58]. In a randomized, open-label, active-controlled, multicenter trial in Italy (MEDITA) [59] and in a meta-analysis [60] based on four randomized clinical trials, low-dose methoxyflurane was shown to have superior efficacy when compared with some analgesics currently used for the treatment of acute musculoskeletal pain associated with trauma. 

The analysis confirmed the rapid onset of pain relief with low-dose methoxyflurane. Improved analgesia was demonstrated on the primary endpoint (difference in pain intensity) from 5 min after treatment initiation and was maintained throughout the 30 min assessment. The good analgesic effect of low-dose methoxyflurane was also consistent across a range of other endpoints, including time to pain relief and various response criteria. The improved pain scales were also supported by higher patient, caregiver and even study researcher satisfaction [60].

**Figure 1:**
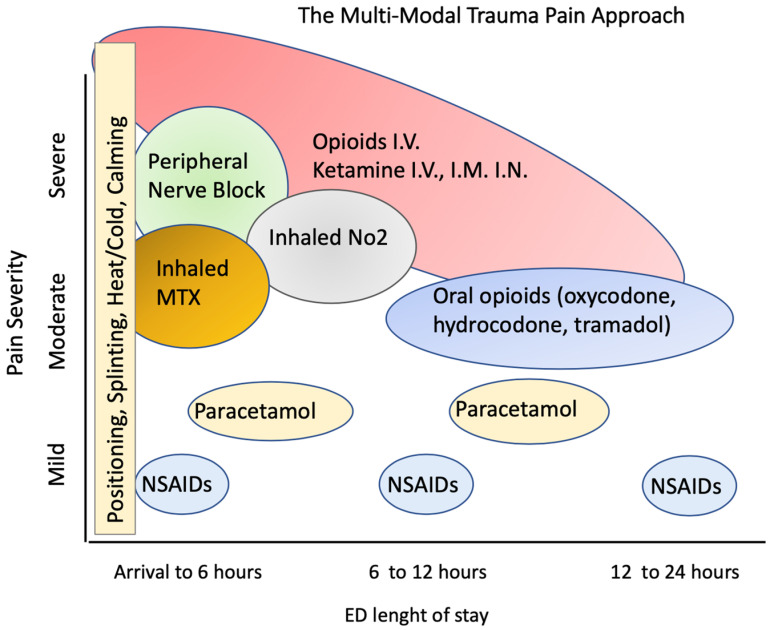
The multimodal approach to trauma patients in relation to the ED pathway from arrival at ED to the final decision in relation to pain severity.

A multimodal pain approach, which involves the use of two or more drugs with different mechanisms of action, plays an important role in the relief of trauma pain [46]. It is defined as the integrated use of multiple strategies that include systemic analgesics, regional analgesic techniques and non-pharmacological interventions to affect peripheral and/or central nervous system sites in the pain pathway [61]. The concept of multimodal analgesia can be applied to the entire treatment continuum, with solutions adapted to each phase of treatment (Figure 1).

The advantages of a multimodal strategy lie in maximizing the different pharmacological mechanisms of the various classes of drugs used in a combination that is useful for pain management (Figure 1). This strategy avoids the use of a single drug class by increasing the dose when the analgesic effect is low. One of the strategies used is to administer non-opioid analgesics (e.g., paracetamol and NSAIDs) on a scheduled basis, rather than as needed, to mitigate the fluctuations between peak and trough serum levels. Drugs such as ketamine and systemic lidocaine are also safe and effective components of a multimodal approach [62].

In a pragmatic randomized trial [63], the widely used combination of oral paracetamol, naproxen, gabapentin, lidocaine patches and opioids (as needed) resulted in a reduction in inpatient opioid burden and opioid prescribing at discharge. 

Outside the area of trauma pain, there is evidence that this approach can reduce the required opioid dose (opioid-sparing effect) [64]. One of the advantages of this strategy is to engage the patient by informing them of the favorable risk-benefit ratio of each component of the treatment plan and that some medications, such as paracetamol and NSAIDs, are not available over the counter but are genuine analgesics [65]. 

Ideally, the trauma pathway should also involve pain specialists, non-pharmacological treatment providers and psychiatrists/staff to ensure a better quality of care for patients with complex pain management needs. 

## 3. The Patient’s Pathway for Trauma Pain in the ED

The ED pathway of a patient who has suffered trauma and is rescued by an ambulance team, accepted in triage, seen by the emergency physician and is subjected to diagnostic tests and treatment for reported injuries must overcome numerous obstacles that unfortunately lead to interruptions and inadequate analgesia throughout his diagnostic and therapeutic path in the ED (Figure 1). These obstacles relate to the limitations of currently available therapies, health professionals’ attitudes towards opioids, lack of validated guidelines for pain management in the ED in most countries and inadequate pain assessment in the emergency setting. To improve trauma pain management and a patient-centered approach, a significant culture change is required among emergency health professionals.

### 3.1. Route of Administration

The specific drug recommended for the treatment of pain in the ED may vary depending on the type of trauma, the severity of the pain, the skill level of the team and the skills and experience of the attending physician [6,13]. A multimodal pharmacological approach is likely to be the most appropriate and thoughtful solution after initial arrival at the ED. 

By integrating different medications with different properties and in different modes of administration, the different pharmacological properties of each medication are enhanced by their use in different combinations, using different mechanisms of action and integrating different effects from arrival at ED until transfer to an ordinary ward or at discharge (Figure 1).

The optimal treatment regimen on arrival at the ED after the use of non-pharmacological interventions, e.g., positioning and splinting of fractures, should include the use of intravenous opiates, ketamine, in combination with discrete amounts of inhaled analgesics and a peripheral block in cases with peripheral or district lesions. This multimodal approach must then be modified in subsequent phases according to the desired outcome, including the possibility of providing effective long-term analgesic treatment, and with drugs such as weak opiates and NSAIDs (Figure 1). 

Figure 1 shows a hypothesis for the pathway of a patient treated with a multimodal pharmacological approach. On arrival, following non-pharmacological treatments, intravenous opiates and ketamine are administered; finally, inhaled analgesia with a mixture of methoxyflurane as an N_2_O alternative is administered. In the phases after the ED, the doses of opioids and ketamine are reduced via the systemic route and other drugs, e.g., weak oral opioids such as oxycodone, hydrocodone and tramadol, supplemented by paracetamol and NSAIDs, are used depending on the final outcome (Figure 1).

The type of analgesics used can have several limitations as a result of their availability and the confidence of individual professionals in their use. The route of administration of analgesics in the management of trauma pain may have several limitations. Intravenous (IV) analgesia is often the most common route of administration in emergency situations and provides rapid onset pain relief [6,13]. However, administration of IV analgesia can be difficult in certain circumstances, such as at the scene of a traffic accident. Difficulties may also arise when attempting to administer IV in cold weather in the prehospital setting or to patients with difficult venous access, causing further inconvenience to the affected individuals and delaying the onset of analgesia. Furthermore, in some countries, e.g., Denmark, many paramedics are not authorized to administer IV medication [66]. 

Intravenous and other routes of drug administration could inflict additional pain on a patient already suffering from trauma pain and are therefore not appropriate in some cases. In addition, administration of some analgesics, while effective in treating pain, might be inappropriate in the presence of significant oedema or hypovolemia, a common condition in severely injured patients [67]. 

The route of intra-muscular administration does not allow for dose titration or adjustment, which may result in ineffective and indeterminate analgesia. In addition, the intraosseous route of administration requires the insertion of an intraosseous needle, which again is painful for the patient and not common in practice. Many injured patients who would be suitable for local anesthesia or regional nerve blocks often do not receive this treatment because the practice is not yet widespread; this is also due to the insufficient competence and experience of surgeons in the use of these techniques [6,13]. 

Intranasal (IN) administration of drugs in trauma is much less invasive than administration via IV, but could be difficult in severe facial trauma, epistaxis, nasal congestion and dysphagia [68]. In these individuals, IN adminstration may result in a suboptimal dose of analgesia and thus ineffective treatment of traumatic pain in the initial phase of treatment (Figure 1).

Recent advances in technology allow continuous regional analgesia that can provide pain relief for several days rather than for hours. Locoregional analgesia provides faster pain relief than systemic analgesia alone, reduces opioid requirements and shortens hospital length of stay [69]. Recent advances in ultrasound-guided regional analgesia have led to the introduction of this method into current clinical practice (Figure 1). These blocks are technically simpler than traditional neuraxial blocks and nerve plexus blocks. These fascial block techniques, apart from the quadratus lumborum block, can be considered acceptable alternatives, even in cases treated with antiplatelet and anticoagulant drugs [70]. 

The development of compartment syndrome in treated cases can be a complication, particularly in cases of limb trauma, especially complex fractures or crush injuries with fragments. Despite the lack of data on this topic, the effect of a complete blockade of sensory and motor function of the limb in these cases may delay appropriate and timely diagnosis of compartment syndrome [71]. To minimize the risk, low doses of analgesics should be used in these cases in order to achieve a partial sensory/motor blockade. 

Regional analgesia requires investment in both the training of professionals and the organization of treatment pathways. Systemic toxicity of local anesthetics is a complication that can occur. Mild manifestations include dizziness, tinnitus and perioral numbness. More severe cases with seizures or even cardiac arrest have also been reported. Systemic toxicity from the local anesthetic may occur immediately during block placement or up to 45 min after completion of the procedure. [72]. The occurrence of systemic toxicity of the local anesthetic can be blocked by the rapid administration of a 20% lipid emulsion [73].

### 3.2. Main Drugs Available

Opioids are considered the cornerstone for the treatment of severe pain in emergency trauma patients.

Opioids have been shown to be very effective but have a problematic safety profile associated with serious cardiovascular events, acute dyspeptic syndrome with nausea and vomiting and ultimately an increased risk of respiratory failure [74]. The side effects associated with opioids are well described. In most patients, they are either transient and resolve as the patient develops tolerance (e.g., nausea and vomiting), or they persist (e.g., constipation); the clinical response varies [75]. Ketamine caused decreased alertness and agitation in 1.5–18% of cases [72]. Decreases in SpO_2_ were observed with fentanyl (mean 0.6%, maximum 16.1% [76]), ketamine (mean 0.4%, maximum 11.5% [77]) and morphine (mean 0.6%, maximum 4.8% [78]).

Overall, oxygenation with assisted ventilation was required in 0.05% of patients treated with ketamine, in 0.02% of patients treated with fentanyl and in 0% of patients treated with morphine. Hyper-salivation was reported in 0.5–3% of cases, mainly in children, but was not clinically relevant [79]. Nausea and vomiting were the main adverse effects of morphine (4.8%), fentanyl (1.5%) and ketamine (0.5%), while hypotension occurred in 1.6% of cases with fentanyl and 0.5% of cases with morphine.

Tolerance to side effects can also be classified as genetically predetermined and present from the first dose or acquired depending on the treatment. Acquired tolerance may be due to a pharmacokinetic factor (e.g., drug metabolism), a pharmacodynamic factor (e.g., up- or down-regulation of opioid receptors) or even a learned response (e.g., patient expectancy may reduce effect over time) [80].

The side effects of opioids may limit treatment, but a multimodal approach that reduces opioid doses may also reduce adverse events (Figure 1). As a result of the associated adverse events of opioids (especially respiratory depression), these patients must be observed over a longer period, especially in a trauma setting, and vital signs must be continuously monitored. In these cases, the organizational effort in terms of both nursing staff and appropriate technology throughout the patient’s stay is considered high [14].

In clinical practice, special attention is paid to the early assessment and rapid management of pain. Basic measures suggested include early assessment of pain in patients with severe trauma using pain rating scales adapted to the patient’s age, developmental level and cognitive functions, and the use of intravenous morphine as the analgesic of first choice [6,13]. 

Some experience has been gained in determining the optimal dosage of morphine. In a single study [72] comparing a medium with a high dose of morphine, no difference was found in the efficacy of pain treatment, i.e., for the different levels of pain, while the high dose showed an increased incidence of side effects such as nausea and loss of consciousness. 

The NICE guidelines [81] recommend generous use of the different doses and dosages according to the tolerability of the drug. The lowest dose (0.10 mg/kg) is the most used dose in current practice; this dose ensures a sufficient level of analgesia in any case. The comparison of morphine vs. intravenous morphine + ketamine (only two published studies [82,83]) suggests the greater efficacy of combination therapy. Morphine was found to have a higher incidence of nausea, while ketamine was found to have a higher risk of loss of consciousness. Morphine use improved patient satisfaction scores but led to no difference in health-related quality of life.

The sedative effect of ketamine could be useful for facilitating limb manipulation during the ED care pathway, reducing opioid consumption and the risk of subsequent post-traumatic stress syndrome, although evidence of efficacy is currently inconsistent [84]. 

In studies comparing the effects of different opioids, ketamine plus morphine was reported to be more effective than morphine alone, producing a significantly faster effect [85]; pain relief after 30 min was comparable in the two published RCTs [78,79,80,81,82,83,84,85,86], but adverse events (breathing problems and vomiting) were more common in the morphine-treated group [87]. The results of the two randomized controlled trials comparing the effect of ketamine/midazolam i.v. with that of fentanyl/midazolam i.v. showed no differences in terms of efficacy in pain management [88], but faster pain reduction in the fentanyl/midazolam group and a lower risk of hypoxia in the ketamine-treated group [89].

When comparing fentanyl with morphine, a retrospective study reported similar efficacy in terms of pain relief, with fentanyl having an advantage over morphine in terms of speed of action [89].

A comparison of morphine + ketamine in one study RCT showed similar efficacy in pain management, while in other studies, ketamine alone or in combination with other substances was more effective and faster acting than morphine alone [85]. The duration of the analgesic effect of these drugs ranges from 10 to 15 min for ketamine, 20 to 40 min for fentanyl and up to 4 h for morphine [90].

The use of IV morphine has also been compared to the use of other drugs, such as IV fentanyl. The results did not document, in the available evidence [66], significant differences in terms of the efficacy of the treatment, i.e., pain relief, and in terms of adverse side effects. A further comparison was considered between the use of IV morphine and IV paracetamol. The results of the studies skew, in terms of analgesic efficacy and in patient satisfaction, in favor of the use of morphine, even if associated with a higher profile of adverse effects [67].

In the NICE guidelines, paracetamol is considered inappropriate as the sole intervention for severe trauma. It has been noted that paracetamol may have a morphine-sparing effect, but that morphine should always be preferred as first-line therapy. 

The efficacy of some weaker analgesics, including metamizole, paracetamol and NSAIDs, in the treatment of trauma pain is limited, especially in cases where pain may increase rapidly during the particular phase, e.g., transport to ED [13]. When deciding to use paracetamol, for example, special attention should be paid to cases of possible overdose if the patient has taken it prior to the phase, as paracetamol is an over-the-counter product [91]. It is noteworthy that a review of observational studies found considerable toxicity of paracetamol at the upper end of standard analgesic doses [92]; however, the focus of the study was not on emergency trauma pain.

Analgesic treatment with NO_2_ has been proposed as an alternative for the emergency treatment of trauma, especially in the initial phase of on-site trauma treatment. Pain relief in these cases, even if achieved quickly, may not be appropriate for some patients, e.g., patients with pneumothorax or head/face trauma. The poor response to treatment in these cases leads to selective use of the drug [93]. Despite its proven analgesic effect, some operational problems at the site of use might hinder the use of N_2_O, as it requires large amounts of equipment with the transport of bulky bottles of premixed N_2_O and oxygen [94]. 

It is also important to consider that the mixture of oxygen and N_2_O can separate at low temperatures and that it must be stored at temperatures above 10 °C for at least 24 h before use to avoid releasing potentially hypoxic concentrations when the cylinder is emptied [95]. The equipment required for treatment with N_2_O could also be a limitation in difficult situations (e.g., remote locations) due to the difficulty of transport in emergency vehicles. In the prehospital setting, there is significant experience with methoxyflurane administered via a portable inhaler. Given the analgesic efficacy of methoxyflurane, the practicality of the device and the ease of use, this solution could overcome some of the limitations associated with treatment with N_2_O, including its particular suitability for use in remote locations or for helicopter rescue, including on behalf of paramedic [94].

### 3.3. Limitations

Insufficient analgesia is due to lack of public health confidence in the use of opioids [95]. For example, in an Italian ED, only 3% of patients received opioids, despite 77% of patients reporting severe pain that warranted opioid therapy according to the centers’ pain management protocol [96]. Healthcare providers’ reluctance to administer opioids has been attributed to legal barriers to prescribing, concerns about patient behavior or the risk of pathological dependence, increased monitoring needs and fears that analgesia masks other trauma symptoms [97,98]. Incidentally, this phenomenon of “aversion to opioids” has been widely documented in large studies [99,100]. 

Since opioids are one of the leading solutions for moderate to severe pain in trauma, aversion to their use can lead to an insufficient number of cases being adequately treated. The unwillingness of medical staff to prescribe and directly administer opioids hinders pain management in both prehospital care and the emergency department. In different settings, some patients also express concerns about opioid treatment and desire non-opioid pain management [101]. 

When used appropriately, opioids are an effective treatment for severe trauma pain. Overcoming physician and patient aversion to opioid use can therefore reduce the burden of trauma pain by providing effective treatment. This could potentially be achieved by the diffusion of national evidence-based treatment guidelines that clearly document the appropriate use of opioid analgesics for short-term administration in order to address acute traumatic pain, including patient selection [6,13]. Readily available information on the risk of side effects such as overdose and/or dependence associated with acute use could also be useful in helping healthcare professionals make informed decisions in traumatic emergency settings, since much of the information in the literature on these issues concerns chronic opioid therapy [102,103].

Rapid pain relief is a fundamental element of the treatment of trauma patients in the ED, as even a minimal delay means unnecessary suffering for the patient [104]. The likelihood of not achieving this goal may be greater in the early stages of ED care and especially during tests and procedures, since a significant proportion of patients with moderate-severe pain after trauma do not receive analgesic treatment in an appropriate time (15–20 min) after entry, as recommended in the main European guidelines [13].

In the ED, the fundamental node for adequate and timely pain management is represented by the assessment of the injured patient at triage. The triage acceptance phase is the fundamental phase for proper assessment and treatment with appropriate pain relief. Incorrect assessment or delay in this step would certainly hinder timely pain relief [105]. 

To date, triage protocols used in the emergency department usually include pain assessment as an essential “vital sign” for timely pain management, especially in trauma patients [5,6,13]. Considering extensive training programs and efforts by emergency medical societies, triage assessment of pain in patients with moderate and severe trauma falls short of expectations. Even the attitude of some health care workers, who consider pain secondary to other impaired vital parameters in emergencies, is certainly a critical element for timely treatment with analgesics, as it is not considered as important an element as blood pressure, respiratory rate or state of consciousness for the patient’s outcome [106]. 

Failure to treat pain in a timely manner in the prehospital phase could lead to greater difficulties for health professionals who must manage the subsequent phases. Indeed, in these cases, the frequent hemodynamic and metabolic fluctuations of the patient could affect both the pharmacokinetics and pharmacodynamics of the drugs and delay the effectiveness of analgesic treatment [107]. 

The short time on scene and the difficulties of subsequent transport in the ambulance rescue would be the main reasons for the lack of effective treatment [106]. It should be noted that in some European countries, ambulance personnel are not authorized to administer opioid analgesics, although a study in Germany supports the importance of prehospital administration of fentanyl and morphine, including by specially trained paramedics [108]. Finally, overcrowding at EDs is also a crucial factor in delaying the treatment of trauma pain, as medical staff are under enormous pressure in this context. The excessive number of patients requiring treatment in the ED can inevitably lead to an increase in patient assessment time and thus treatment time [109]. 

## 4. Conclusions

Considering the evidence from the published literature, the management of moderate to severe trauma pain in the ED could be improved by increasing the use of pain rating scales and developing and implementing effective pain management protocols, especially with a multimodal drug approach. These measures will reduce healthcare costs for inadequately treated patients in emergency situations. 

The use of an increasing number of analgesics, a greater number of different delivery devices and a proliferation of available medications will allow both the cultural and professional barriers that currently affect the outcome of these patients to be overcome.

## Figures and Tables

**Table 1:** The main pain ranking scales in the evaluation of subjects with trauma in different age categories.

Age Category	Scale	Ranking	Interpretation
0 to 4 years	Faces, Arms, Legs, Cry, Consolability (FLACC) [19,20]	0 to 2 for each item Score from 0 to 10	0 relaxed-comfortable 1 to 3 mild discomforts 4 to 6 moderate 7 to 10 severe
0 to 4 years	Wong–Baker Faces^®®^ [21]	Six different faces (6 scores)	
Over 4 years	Numerical Rating Scale (NRS) [22]	0: no pain 1 to 3 mild 4 to 6 moderate 7 to 10 severe	
Adults and Older	Visual Analogue Scale: (VAS) [23]	A 100 mm horizontal line with no pain written on the left and worst possible pain on the right side	
	Numerical Rating Scale (NRS) [22]	0: no pain 1 to 3 mild 4 to 6 moderate 7 to 10 severe	
Older	Pain Assessment in Advanced Dementia (PAINAD) [24]	Breathing, negative vocalization, facial expression, body language, consolability Score from 0 to 3 for each item 5 items considered	0 to 3 mild 4 to 6 moderate 7 to 10 severe

## Data Availability

Not applicable.
